# Neonatal melioidosis case reports—Lessons learned

**DOI:** 10.1002/ccr3.2584

**Published:** 2019-12-03

**Authors:** Sylvia Daim, Ester Barnad, Victor Johnny, Maria Suleiman, Muhammad Jikal, Tock Hing Chua, Christina Rundi

**Affiliations:** ^1^ Department of Pathobiology and Medical Diagnostics Faculty of Medicine and Health Sciences Universiti Malaysia Sabah Kota Kinabalu Malaysia; ^2^ Sabah State Health Department Kota Kinabalu Malaysia

**Keywords:** *Burkholderia pseudomallei*, ceftazidime, melioidosis, neonatal, Sabah Malaysia

## Abstract

In endemic regions, include melioidosis in the routine differential diagnosis of neonates with respiratory distress, and consider early empirical ceftazidime treatment for neonates with worsening respiratory distress.

## Graphical abstract

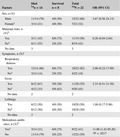

## 1 INTRODUCTION

Three key lessons were identified from the review of 22 case reports: (a). In endemic regions, melioidosis should be included in the routine differential diagnosis of neonates with respiratory distress; (b). Early empirical ceftazidime treatment may need to be considered for neonates with worsening respiratory distress; (c). Clinicians are to be routinely updated of the local melioidosis prevalence.

Melioidosis is typically a community‐acquired infectious disease caused by the soil bacterium, *Burkholderia pseudomallei*. In endemic regions where the diagnostic capability and capacity are limited, disease mortality could exceed 70%. Clinical spectrum is complex and wide‐ranging, making melioidosis generally challenging to diagnose and treat. Main transmission mode is via direct contact with or ingestion of contaminated water or soil, or inhalation of contaminated dust particles.^1^ Incidence of neonatal melioidosis, therefore, tends to be rare even in endemic regions.^2^, ^3^ New cases and their clinical reports are thus valuable, as they lend additional clinical and epidemiological data that could improve our understanding of the disease in neonates. We report here two new cases from Sabah, Malaysia, and further reviewed published case reports available to date to have a better grasp of the factors that affect disease outcome in neonatal melioidosis.

## 2 CASE 1

A term baby boy weighing 3100 g was born with good Apgar scores via spontaneous vaginal delivery (SVD) to a G2P1, healthy mother. He was discharged home well on the same day. At 16 days of age, he attended a clinic with a 3‐days history of cough, runny nose, and fever. Tachypnoea was noted with crepitation heard over the lungs. He was given nebulized salbutamol and discharged with sirup paracetamol. At 17 days of age, he was admitted to the district hospital with rapid breathing and lethargy being the chief complaints. Bilateral lung crepitation was detected. No subcostal and intercostal retractions were observed. Chest X‐ray showed bilateral pneumonic changes. Provisional diagnosis was bronchopneumonia, and he was given intravenous penicillin (50 000 unit/kg) and gentamicin (5 mg/kg) and placed on headbox oxygen therapy. On day three postadmission, he still had spiking fever (38°C) despite antibiotic treatment. He also developed frequent desaturations <85%, requiring increased headbox oxygen from 5 to 8 L/min, as well as bilateral crepitation with occasional deep subcostal retractions. He was then transferred to the referral hospital. On admission, he was tachypneic with retraction and was put on nasal continuous positive airway pressure (CPAP) and given cefuroxime. At 6 hours postadmission, he was increasingly tachypneic with deeper retractions, requiring respiratory support with FiO_2_ > 50%. Desaturations <90% became frequent, and he was intubated. Chest X‐ray revealed acute respiratory distress syndrome‐like picture. Antibiotics were escalated to cefepime and cloxacillin. Saturation was unable to be maintained, and ventilation was escalated to high‐frequency oscillatory ventilation (HFOV) at 12 hours postadmission. The highest SpO_2_ achieved was 85%‐88%. He also developed recurrent hypoglycemia and persistent metabolic acidosis (pH 7.0‐7.1). At 26 hours postadmission, he was in asystole and succumbed at 22 days of age. Blood samples on admission at the district hospital were negative for microbial growth, while samples on admission at the referral hospital were positive for *B pseudomallei* growth. Cerebrospinal fluid (CSF) sampled at postmortem was also positive for *B pseudomallei* growth. The isolate was susceptible to meropenem, tetracycline, and trimethoprim/sulfamethoxazole, but resistant to amoxicillin/clavulanic acid.

## 3 CASE 2

A baby girl weighing 2500 g was born at 37 weeks via SVD to a 34‐year‐old, G7P6 mother, who was anemic during the pregnancy. The baby was initially breeched, and emergency lower segment cesarean section had been planned, but the mother went into spontaneous labor. Meconium‐stained liquor was noted. Apgar score was 6 at 1 minute, which improved to 10 at 5 minutes after oral‐nasal suction was performed. The baby was mildly tachypneic with minimal retraction and required headbox oxygen therapy for 30 minutes. She was discharged home at 2 days of age, but suffered persistent jaundice from 3 days of age. On day 4, she was admitted to the clinic for 4 days and given phototherapy for the jaundice. At 22 days, she was found limp, cyanotic, and apneic when returning to the clinic for serum bilirubin review. After resuscitation, she was transferred and admitted to the district hospital. Chest X‐ray on admission revealed a consolidation on the right midzone. Provisional diagnosis was aspiration pneumonia, and intravenous penicillin (50 000 unit/kg) and gentamicin (5 mg/kg) were initiated. She was also put on nasal prong oxygen (NPO_2_) therapy. However, she developed worsening respiratory distress, and by 12 hours postadmission, she was cyanotic again. Bilateral lung crepitation was detected, and saturation under NPO_2_ was unable to be maintained. She was subsequently intubated. Chest X‐Ray postintubation showed worsening bilateral consolidation, and antibiotic was empirically upgraded to cefotaxime and penicillin to cover for potential meningitis. She was then transferred to the referral hospital's level 3 neonatal intensive care unit (NICU). Nevertheless, her progress was unsatisfactory for the past 40 hours of admission: Persistent metabolic acidosis (pH6.98 on admission and pH7.26 at 16 hours postadmission), multiple desaturations to <70% leading to trial of HFOV at 36 hours postadmission, repeated chest X‐ray at 10, and 36 hours postadmission still showed dense bilateral consolidations, and two episodes of jerky movements over bilateral upper limbs were noted at 12 and 32 hours postadmission. At 31 hours postadmission, her blood culture revealed gram‐negative rods, and antibiotic was escalated to cefepime. She became bradycardic, down to 40 beats per minute at 38 hours postadmission despite on triple inotropes. In view of the poor progress, the parents requested withdrawal of care. She was pronounced dead 2 hours later at 24 days of age (40 hours postadmission). Blood samples on admission at the district hospital were negative for microbial growth, while samples on admission at the referral hospital were positive for *B pseudomallei* growth. CSF was managed to be sampled only at postmortem and was also positive for *B pseudomallei* growth. The isolate was susceptible to amoxicillin/clavulanic acid, ceftazidime, imipenem, meropenem, tetracycline, and trimethoprim/sulfamethoxazole.

## 4 DISCUSSION

Neonatal melioidosis is considered a rare infection even in endemic regions.^2^, ^3^ The only children hospital in Sabah, for example, had only two confirmed cases on record in year 2000‐2012.^4^ In another example, out of the 59 cases of confirmed pediatric melioidosis admitted to Hatyai Hospital between 1985 and 1998, only eight were neonatal cases.^5^ Clinical reports of new cases are thus valuable as they could add on to existing clinical data and strengthen the clinical evidences concerning the disease.

In a previous systematic review of the literature, 22 neonatal melioidosis cases have been identified with primary outcome, which is survival of the neonates from the infection.^6^ Since then, six more neonatal melioidosis cases that have primary outcome were published,^4^, ^7^, ^8^, ^9^, ^10^ and including the two cases presented here, the number of cases available for review totaled to 30. However, detailed clinical data were not available for eight cases, all of which came from a case series.^5^, ^6^ Of the 30 cases, eight are from Malaysia.^4^, ^11^, ^12^, ^13^ Survival rate of the neonates were found very low, with case fatality rate at 73% (22/30). This was still much higher compared with childhood and adult melioidosis, in which fatality rates ranged at 7 ~ 60%.^1^, ^2^, ^3^, ^14^

Ceftazidime or meropenem is the currently recommended antibiotics to treat acute‐phase melioidosis.^1^ The two cases reported here did not receive either antibiotics, and so was a large proportion of the previously reported cases (59%; 13/22; Table 1). The lack of melioidosis antibiotic treatment was found to be the most significant factor contributing to a high case fatality rate at 79% (11/14, *P* = .0217; Table 1). The odds for fatal outcome were 11.00 times higher among neonates who did not receive melioidosis antibiotics than the odds were among those who did. Adherence to usage guidelines^15^ coupled with slow diagnosis may have resulted in melioidosis antibiotics not being given to these patients. Usage of ceftazidime or meropenem usually requires a high level of clinical suspicion and supported by microbiology results and local disease prevalence.

**Table 1:** Factors affecting neonatal melioidosis outcome

Factors	Died 5, 6n = 14	Survived n = 8	Total 5, 6N = 22	OR (95% CI)
Sex, n (%)				
Male	11/14 (79)	4/8 (50)	15/22 (68)	3.67 (0.56‐24.13)
Female^a^	3/14 (21)	4/8 (50)	7/22 (32)	
Maternal risks, n (%)^b^				
Yes	5/11 (45)	6/8 (75)	11/19 (58)	0.28 (0.04‐2.04)
No^a^	6/11 (55)	2/8 (25)	8/19 (42)	
No data	3		3	
Symptoms, n (%)^c^				
Respiratory distress				
Yes	12/14 (86)	6/8 (75)	18/22 (82)	2.00 (0.22‐17.89)
No^a^	2/14 (14)	2/8 (25)	4/22 (18)	
Fever				
Yes	8/12 (67)	3/8 (38)	11/20 (55)	3.33 (0.51‐21.58)
No^a^	4/12 (33)	5/8 (63)	9/20 (45)	
No data	2		2	
Lethargy				
Yes	6/12 (50)	4/8 (50)	10/20 (50)	1.00 (0.17‐5.98)
No^a^	6/12 (50)	4/8 (50)	10/20 (50)	
No data	2		2	
Melioidosis antibiotics, n (%)^d^				
Yes^a^	3/14 (21)	6/8 (75)	9/22 (41)	11.00 (1.42‐85.20) * *P* < .0217
No	11/14 (79)	2/8 (25)	13/22 (59)	

Note

Eight cases with primary outcome (all fatal) but no clinical data were excluded.^5^, ^6^

Abbreviations: CI, confidence interval; OR, odds ratio.

a Reference groups.

b Includes premature rupture of membrane (PROM), preeclampsia, placenta praevia, chorioamnionitis, and maternal infections.

c Onset symptoms.

d Ceftazidime or meropenem.

* Fisher's exact test.

At present, melioidosis diagnosis still relies mainly on the capability and capacity to culture *B pseudomallei* from clinical samples. This imposes a delay in initiating timely melioidosis antibiotic treatment on the patients. An average of 48 hours is needed to grow *B pseudomallei* in microbiological cultures, followed by another 24‐48 hours of biochemical identification processes. The delay could be further compounded if patient samples needed to be sent to another facility for diagnosis. In the two cases reported here, the peripheral hospitals do not have the capacity to microbiologically diagnose melioidosis, and the patient samples were sent to the nearest tertiary hospitals. Exact causes of negative growth in the cultures of blood samples collected while at the peripheral hospitals were not known. Great reduction in the number of culturable *B pseudomallei* during processing and delivery of the blood samples offered one plausible explanation. In the time taken to culture *B pseudomallei* from their blood samples, the two neonates did not respond to penicillin, gentamicin, and other subsequent antibiotics. At the same time, their clinical conditions rapidly deteriorated, both manifesting chiefly as worsening respiratory distress. They died before microbiological diagnosis can be completed.

The three most frequent clinical manifestations of neonatal melioidosis presented at hospitals were respiratory distress (82%; 18/22) typically dyspnea, tachypnea, or apnea, followed by fever (55%; 11/20), and lethargy (50%; 10/20). A high proportion of the fatal cases had some form of respiratory distress (86%; 12/14). These are nonspecific symptoms, also commonly manifested in other neonatal infections caused by either bacteria, viruses, and fungi.^16^ Consequently, melioidosis can be easily overlooked and excluded from routine differential diagnosis. Only clinicians who are familiar with the disease will be able to empirically initiate early melioidosis antibiotic treatment. Given the high case fatality rate associated with the lack of melioidosis antibiotic treatment (79%), consideration to initiate early empirical ceftazidime or meropenem even before microbiological diagnosis is known, may need to be given to neonates in endemic regions, especially those who manifest worsening respiratory distress and concurrently fail to respond to penicillin and gentamicin.

The main transmission mode of *B pseudomallei* is via direct contact with or ingestion of contaminated water or soil, or inhalation of contaminated dust particles.^1^ In neonates, the routes of infection become ambiguous especially, if evidences of direct exposures were lacking. Cases of mother‐to‐child transmission have been documented, but these are extremely rare.^17^, ^18^ The Netherland case was an early‐onset neonatal sepsis, and the likely infection route suggested is via the placenta.^17^ The Australian case also was an early‐onset neonatal infection, in which the transmission route suggested is via the mother's breast milk.^18^ In the two cases reported here, evidences were lacking to support for a mother‐to‐child transmission. First, both mothers claimed to have no history of infections during pre‐ and postpartum period. The respective clinics and hospitals also did not observe evidence of infections in the mothers. Secondly, the symptoms in the neonates were late‐onset, at day 16 and day 22 of age for Case 1 and Case 2, respectively. In the two cases reported here, inhalation of contaminated dust particles became a probable route of infection, as evidences for direct contact with, or ingestion of contaminated water or soil also were not forthcoming.

A male‐biased incidence was found among the neonatal melioidosis cases (68%; 15/22), similar to childhood^3^, ^4^, ^19^ and adult melioidosis.^14^, ^20^, ^21^, ^22^ The odds of fatal outcome were found 3.67 times higher among male than the odds were among female neonates. Association of the male sex with fatal neonatal melioidosis although was not statistically significant (*P* = .2127), there could be an underlying biological significance given the consistently reported higher incidence in male across the age groups. Other infectious diseases that demonstrate higher incidence in male include syphilis, tuberculosis, influenza, hepatitis, and leishmaniasis.^23^ Future study is needed to determine if there were sex‐specific biological factors that affect human immune response and susceptibility to *B pseudomallei* infection as the epidemiological evidences appear to implicate. A neglect of study in this area could potentially cause opportunity to be missed for the development of improved melioidosis treatment methods that take into account patients' sex‐specific biological factors.

## 5 CONCLUSIONS

In endemic regions, melioidosis is recommended to be included in the routine differential diagnosis of neonates who manifest any form of respiratory distress, particularly dyspnea, tachypnea, or apnea. Furthermore, it may be prudent to consider pneumonia within the sepsis spectrum since the clinical signs and organ involvement overlap.^24^ Early empirical ceftazidime treatment may need to be considered in neonates with worsening respiratory distress, who concurrently fail to respond to penicillin and gentamicin. Finally, health departments should regularly promote awareness of the disease and its prevalence among clinicians in their jurisdiction through whatever practical and effective means of communications available.

## CONFLICT OF INTEREST

All authors declare no conflict of interest.

## AUTHOR CONTRIBUTIONS

SD: compiled data from previously published cases, reviewed the cases, analyzed the data, interpret the findings, and wrote the paper with input from all authors. EB and VJ: collected and compiled the data on the two new cases. MS, MJ, and CR: contributed data on the two new cases. THC aided with data analysis and interpretations.
